# Quantitative magnetic resonance imaging of the upper trapezius muscles – assessment of myofascial trigger points in patients with migraine

**DOI:** 10.1186/s10194-019-0960-9

**Published:** 2019-01-18

**Authors:** Nico Sollmann, Nina Mathonia, Dominik Weidlich, Michaela Bonfert, Sebastian A. Schroeder, Katharina A. Badura, Tabea Renner, Florian Trepte-Freisleder, Carl Ganter, Sandro M. Krieg, Claus Zimmer, Ernst J. Rummeny, Dimitrios C. Karampinos, Thomas Baum, Mirjam N. Landgraf, Florian Heinen

**Affiliations:** 1 Department of Diagnostic and Interventional Neuroradiology, Klinikum rechts der Isar, Technische Universität München, Ismaninger Str. 22, 81675 Munich, Germany; 2 TUM-Neuroimaging Center, Klinikum rechts der Isar, Technische Universität München, Munich, Germany; 3 Department of Pediatric Neurology and Developmental Medicine and LMU Center for Children with Medical Complexity, Dr. von Hauner Children’s Hospital, LMU – University Hospital, Ludwig-Maximilians-Universität, Lindwurmstr. 4, 80337 Munich, Germany; 4 Department of Diagnostic and Interventional Radiology, Klinikum rechts der Isar, Technische Universität München, Ismaninger Str. 22, 81675 Munich, Germany; 5 Department of Neurosurgery, Klinikum rechts der Isar, Technische Universität München, Ismaninger Str. 22, 81675 Munich, Germany

**Keywords:** Magnetic resonance imaging, Migraine, Myofascial trigger points, Trapezius muscle, T2 mapping, Trigemino-cervical complex

## Abstract

**Background:**

Research in migraine points towards central-peripheral complexity with a widespread pattern of structures involved. Migraine-associated neck and shoulder muscle pain has clinically been conceptualized as myofascial trigger points (mTrPs). However, concepts remain controversial, and the identification of mTrPs is mostly restricted to manual palpation in clinical routine. This study investigates a more objective, quantitative assessment of mTrPs by means of magnetic resonance imaging (MRI) with T2 mapping.

**Methods:**

Ten subjects (nine females, 25.6 ± 5.2 years) with a diagnosis of migraine according to ICHD-3 underwent bilateral manual palpation of the upper trapezius muscles to localize mTrPs. Capsules were attached to the skin adjacent to the palpated mTrPs for marking. MRI of the neck and shoulder region was performed at 3 T, including a T2-prepared, three-dimensional (3D) turbo spin echo (TSE) sequence. The T2-prepared 3D TSE sequence was used to generate T2 maps, followed by manual placement of regions of interest (ROIs) covering the trapezius muscles of both sides and signal alterations attributable to mTrPs.

**Results:**

The trapezius muscles showed an average T2 value of 27.7 ± 1.4 ms for the right and an average T2 value of 28.7 ± 1.0 ms for the left side (*p* = 0.1055). Concerning signal alterations in T2 maps attributed to mTrPs, nine values were obtained for the right (32.3 ± 2.5 ms) and left side (33.0 ± 1.5 ms), respectively (*p* = 0.0781). When comparing the T2 values of the trapezius muscles to the T2 values extracted from the signal alterations attributed to the mTrPs of the ipsilateral side, we observed a statistically significant difference (*p* = 0.0039). T2 hyperintensities according to visual image inspection were only reported in four subjects for the right and in two subjects for the left side.

**Conclusions:**

Our approach enables the identification of mTrPs and their quantification in terms of T2 mapping even in the absence of qualitative signal alterations. Thus, it (1) might potentially challenge the current gold-standard method of physical examination of mTrPs, (2) could allow for more targeted and objectively verifiable interventions, and (3) could add valuable models to understand better central-peripheral mechanisms in migraine.

## Background

Migraine belongs to the primary headaches, representing the sixth most disabling disorder worldwide, with number-1-status in the age group between 15 to 49 years of age, and affecting about 16% of the European population [1, 2]. To date, much attention has been paid to central mechanisms of migraine, including, but not limiting research to the activation of the trigemino-vascular system [3, 4]. However, it becomes more and more evident that headache may be linked to nociceptive inputs from peripheral structures that can converge upon the same bipolar neurons, with pain from the pericranial head or the neck and shoulder region being referred to the brainstem and meninges and being experienced as headache [5–8]. Thus, research on the development and maintenance of primary headache increasingly points towards a widespread pattern including structures also beyond the central nervous system, with neck and shoulder muscle pain, clinically often represented by the presence of myofascial trigger points (mTrPs), getting in the focus [5, 6, 9, 10].

Such mTrPs are regarded as hyperirritable spots associated with a taut band of skeletal muscle, reacting painful on compression or stretch, and leading to typical referred pain patterns [5–8]. Indeed, muscular pain in the neck and shoulder area has shown to be particularly common in subjects suffering from migraine, with referred pain patterns from mTrPs in neck and shoulder muscles potentially contributing to migraine symptoms [8, 10]. Studies have repeatedly demonstrated a high occurrence of mTrPs in subjects with migraine and provided evidence of associations between such points and neck mobility [11–15]. The link of mTrPs of the neck area to migraine is further suggested by investigations that were successful to provoke migraine attacks by manual palpation delivered specifically to these points [12, 16]. However, despite the interest in mTrPs, reliable detection and characterization by means of verifiable imaging lacks behind. Accordingly, the current gold standard for detection of mTrPs is still represented by manual palpation of muscles, thus being basically unchanged since decades [17, 18]. The approach of such physical examination is questioned with respect to reproducibility and reliability though, with examiners potentially showing considerable disagreement during the diagnosis of mTrPs [19, 20]. Hence, manual palpation is limited due to missing objective verifications, making controlled studies difficult or even impossible.

Efforts have been undertaken to come up with more reliable methods for the identification of mTrPs, including infrared thermography (IT), needle electromyography (EMG), ultrasound (US) including elastography, and also magnetic resonance imaging (MRI) [21–30]. Studies using IT have shown to not agree on skin temperature patterns in the presence of mTrPs [31]; furthermore, the technique seems not widely available. EMG and US have shown more promising results, but needle EMG is invasive and does not directly visualize mTrPs whilst US has demonstrated inconclusive results and commonly provides mere qualitative, descriptive data in the sense that mTrPs might be registered as hypoechoic regions during US examinations [23–26, 29, 30]. To date, MRI has only been applied in few studies for the purpose of identifying mTrPs, with the focus on qualitative image assessments and evidence being limited due to poor agreement between physicians and raters [21, 22]. However, MRI is characterized by superior soft tissue contrast and principally allows for discrimination of even small soft tissue changes when applied with high resolution, thus suggesting high potential for the evaluation of mTrPs in general. Furthermore, there are now MRI-based techniques at hand that enable sensitive quantitative, thus more objective assessments of the body’s musculature even in geometrically complex areas such as the trapezius muscles [32].

Given this background, the present study applies high-resolution, quantitative MRI by means of T2 mapping for the identification of mTrPs in subjects with migraine. Specifically, we aim to assess whether there are quantitatively assessable signal alterations in the muscle area of clinically detectable mTrPs, which are not registered by the eye of a radiologist during mere qualitative image interpretation.

## Methods

### Ethics

The study was approved by our local ethics committee (registration numbers: 154–12 & 5679/13) and conducted in accordance with the Declaration of Helsinki. Written informed consent was obtained from all participants.

### Participants and design

Ten subjects with clinically confirmed migraine were enrolled. These subjects represent a subsample derived from another study that included assessments of mTrPs, with all included subjects having a confirmed history of uni- or bilateral mTrPs within the upper trapezius muscles. Subjects were recruited via an official advertisement on the websites of the two Munich universities, which included a short description of the study’s setup and goals.

All subjects first underwent physical examination of the trapezius muscles bilaterally to identify and localize mTrPs, followed by high-resolution MRI during the same appointment. The subjects were instructed to present totally recovered to the study appointment (no physical activity, no passive or active physiotherapy or yoga on the same or previous three days). The inclusion criteria for this study were 1) written informed consent, 2) age above 18 years, 3) diagnosis of migraine (according to ICHD-3), and 4) reported mTrPs within the upper trapezius muscles. Exclusion criteria were 1) any history of neurological disorders (except for migraine), 2) pregnancy, and 3) general contraindications for MRI (e.g., cochlear implants). After the appointment including physical examination and high-resolution MRI, no follow-up examinations were performed in the context of the present study.

### Physical examination

A certified physiotherapist performed bilateral manual palpation of the upper trapezius muscles with the aim to localize one active mTrP per side. During physical examination, (1) a tender spot within a palpable taut band of muscle fibers had to be palpable, (2) the palpation of the identified structure had to lead to referred cranial pain in typical location for the individual subject, and (3) palpation of the identified structure had to result in a spontaneous defensive movement of the subject (jump sign) to qualify as an active mTrP [6, 21, 33, 34]. Two nitroglycerin capsules were attached to the skin adjacent to an identified active mTrP, with each mTrP being localized in the theoretical connecting line between the respective capsules.

In case of the presence of more than one active mTrP within the upper trapezius muscle per side, the point with the highest intensity of referred cranial pain in typical location was chosen. In case that the physiotherapist only identified an unilateral active mTrP, two capsules where placed contralaterally at a pressure-dolerant point, with this point strictly not fulfilling the criteria of an active mTrP.

### Magnetic resonance imaging

Imaging was performed in supine position using a 3 T whole-body MRI scanner (Ingenia Elition, Philips Healthcare, Best, The Netherlands) in combination with a 16-channel anterior coil, a 12-channel posterior coil, and a 16-channel head coil. The interval between physical examination and MRI was 45 to 60 min on average. Two sequences were acquired after initial survey scanning:

- T2-weighted DIXON turbo spin echo (TSE) sequence: repetition time (TR) / echo time (TE) = 7000 / 100 ms, field of view (FOV) = 474 × 200 × 84 mm^3^, acquisition voxel = 1.8 × 1.8 × 1.8 mm^3^, reconstruction voxel = 0.9 × 0.9 × 1.8 mm^3^, scan time = 5 min 50 s.
- T2-prepared, three-dimensional (3D) TSE sequence [32]: TR / TE = 1500 / 15 ms, FOV = 480 × 200 × 84 mm^3^, acquisition voxel = 2.5 × 2.5 × 3.0 mm^3^, reconstruction voxel = 1.7 × 1.7 × 3.0 mm^3^, echo train length = 55, echo spacing = 2.2 ms, parallel imaging with reduction factor R = 2 × 1.35 (RL × FH), no partial Fourier, fat suppression using spectral inversion recovery, scan time = 7 min 17 s.

During the T2-prepared, 3D TSE sequence, we used a T2 preparation duration of 15-30-45 ms. The flip angle train was determined according to the vendor’s routines for 3D TSE flip angle calculation, leading to a constant signal over the entire shot for the relaxation properties of skeletal muscle [32].

### Generation of T2 maps and evaluation of imaging data

**Fig. 1:**
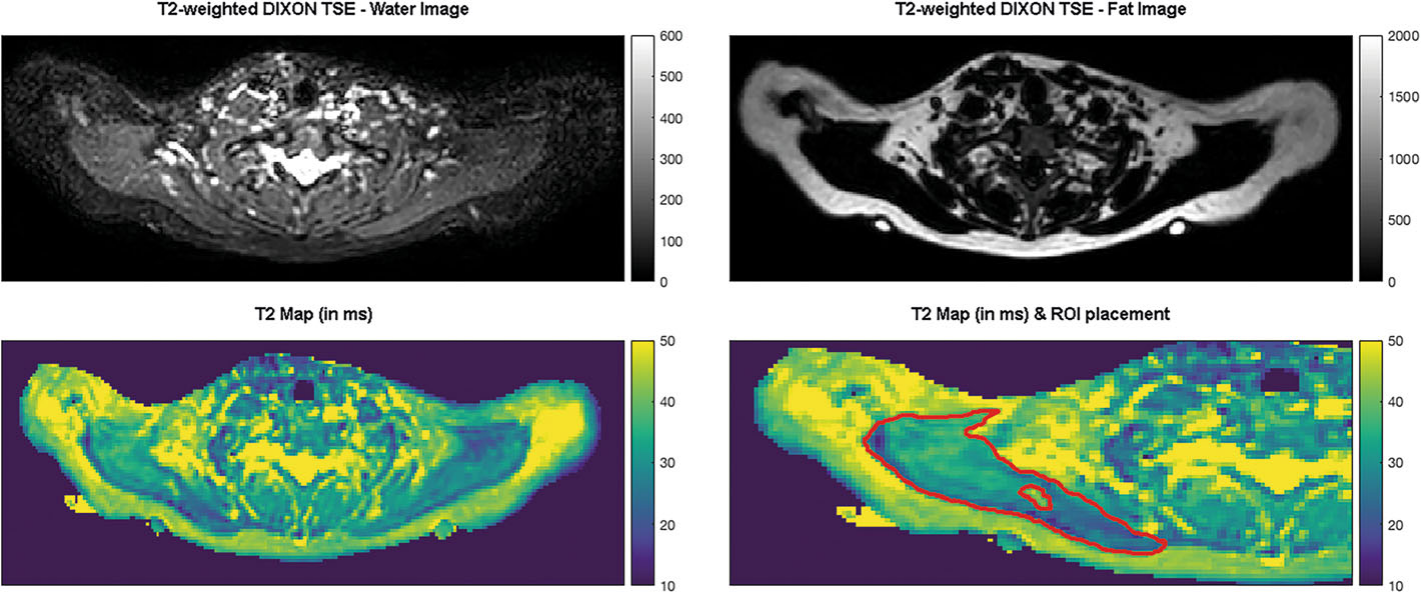
Magnetic resonance imaging (MRI) including T2 mapping of the upper trapezius muscles. This figure captures an exemplary case by showing representative axial slices of the T2-weighted DIXON turbo spin echo (TSE) sequence (upper row). The left upper corner shows the T2-weighted DIXON TSE water image, the right upper corner captures the T2-weighted DIXON TSE fat image. Furthermore, T2 maps as derived from the T2-prepared TSE sequence are displayed (lower row). The left lower corner pictures the color-coded T2 map, the right lower corner shows the same color-coded T2 map after manual placement of regions of interest (ROIs) in the right upper trapezius muscle and with respect to a signal alteration (T2 elevation) within the muscle. In this exemplary case, the signal alteration was located in the area of a manually defined myofascial trigger point (mTrP), as indicated by the spatial relation to superficially attached nitroglycerine capsules as markers. The signal alteration in terms of the circumscribed T2 elevation shows a T2-hyperintense correlate in the T2-weighted DIXON TSE water image

To generate T2 maps out of the T2-prepared, 3D TSE sequence, a voxel-by-voxel approach with a combination of variable projection and golden section search was applied [32, 35, 36]. The T2 maps and the T2-weighted DIXON TSE sequence of each subject were uploaded to Horos software together (version 1.1.7; https://www.horosproject.org), followed by co-registration of the sequences and color coding of the T2 maps (Fig. 1).

In all subjects, the investigator first identified the attached nitroglycerin capsules and re-angulated the images to be able to visualize the capsules in-plane using multi-planar reconstruction (MPR; Fig. 1). This was done using the T2-weighted DIXON TSE sequence, with the investigator being strictly blinded to the results of physical examination regarding the presence of a mTrP or only pressure-dolerant point at the area of the tags. To extract T2 values (in ms), manual placement of regions of interests (ROIs) was then performed using the co-registered and color-coded T2 maps. The axial slice in MPR mode at the level of the capsules was used to carefully draw a polygonal ROI covering the trapezius muscle of each side, respectively (Fig. 1). A margin of approximately 5 mm to the outer contour of the trapezius muscles was considered during ROI placements to avoid the accidental inclusion of muscular fascia or intermuscular fat (Fig. 1). Then, one further ROI was drawn in the color-coded T2 maps on the theoretical connecting line between the capsules per side only in case that the investigator identified a circumscribed signal alteration, being attributable to a mTrP (Fig. 1). The adjacent slices were carefully checked in the color-coded T2 maps and the T2-weighted DIXON TSE sequence, providing better anatomical resolution, to exclude unwilling inclusion of signal alterations due to vessel structures. In case that no signal alteration attributable to a mTrP was observed, no further ROIs were drawn after definition of the ROIs delineating the trapezius muscles.

Furthermore, the color-coded T2 maps were re-angulated to visualize the entire upper trapezius muscle horizontally and in-plane when considering axial slices using MPR, with the supposed mTrP being centered. This was followed by linear measurement of the length of the muscle from the origin to the insertion, which was achieved separately for both sides. An additional linear measurement was performed for each side between the muscle insertion and the signal alterations being attributed to mTrPs, if any. Moreover, the T2-weighted DIXON TSE sequence was qualitatively evaluated to detect any T2 hyperintensities at the area where a signal alteration attributed to a mTrP was identified in the color-coded T2 maps (Fig. 1). This was achieved by careful visual image inspection by the same investigator.

### Statistical analyses

All statistical data analyses and generation of graphs were performed using GraphPad Prism (version 6.0, GraphPad Software Inc., La Jolla, CA, USA). A *p*-value < 0.05 was defined as statistically significant (two-sided).

Descriptive statistics including mean, standard deviation (SD), median, minimum, and maximum values or absolute and relative frequencies were calculated. Regarding the T2 values and length measurements, we separately analyzed the values derived from the left and right side. Shapiro-Wilk normality test indicated non-normally data distribution for the obtained T2 values. We performed Wilcoxon matched-pairs signed rank tests between the T2 values extracted from the left and right trapezius muscles, the left and right signal alterations attributed to mTrPs, the left trapezius muscles and left-sided signal alterations attributed to mTrPs, and the right trapezius muscles and right-sided signal alterations attributed to mTrPs.

## Results

### Cohort characteristics and physical examination

We included ten right-handed subjects (nine females & one male volunteer, mean age: 25.6 ± 5.2 years, range: 19.4–34.9 years), all diagnosed with migraine according to ICHD-3, who reported on migraine since 13.0 ± 9.1 years on average (range: 1.5–29.2 years). The age of the first manifestation of migraine was 12.6 ± 6.5 years (range: 5.0–24.0 years), and subjects suffered from migraine on 5.5 ± 2.4 days per month (range: 3.0–10.0 days per months) with an average pain rating for migraine attacks of 7.2 ± 0.7 points (range: 6.0–8.0 points) according to a numeric pain rating scale from 0 to 10 points. None of the included subjects had any history of neurological disorders (except for migraine). Registered comorbidities of the study cohort were pollen allergy (one subject), asthma (one subject), and hypothyroidism (one subject). Nine of the included subjects regularly performed endurance sports, seven subjects further stated that they regularly participated in strength sports.

MRI was performed in an inter-ictal period in all subjects, with an interval between the last migraine attack and the study appointment of 0.7 ± 1.5 months (range: 0.1–5.1 months). According to physical examination, a clear mTrP was detected in nine subjects within the right-sided trapezius muscle, whereas seven subjects showed a mTrP in the left-sided trapezius. There were no subjects without mTrPs according to manual palpation.

### Imaging of the upper trapezius muscles

#### Measurement of T2 values

**Fig. 2:**
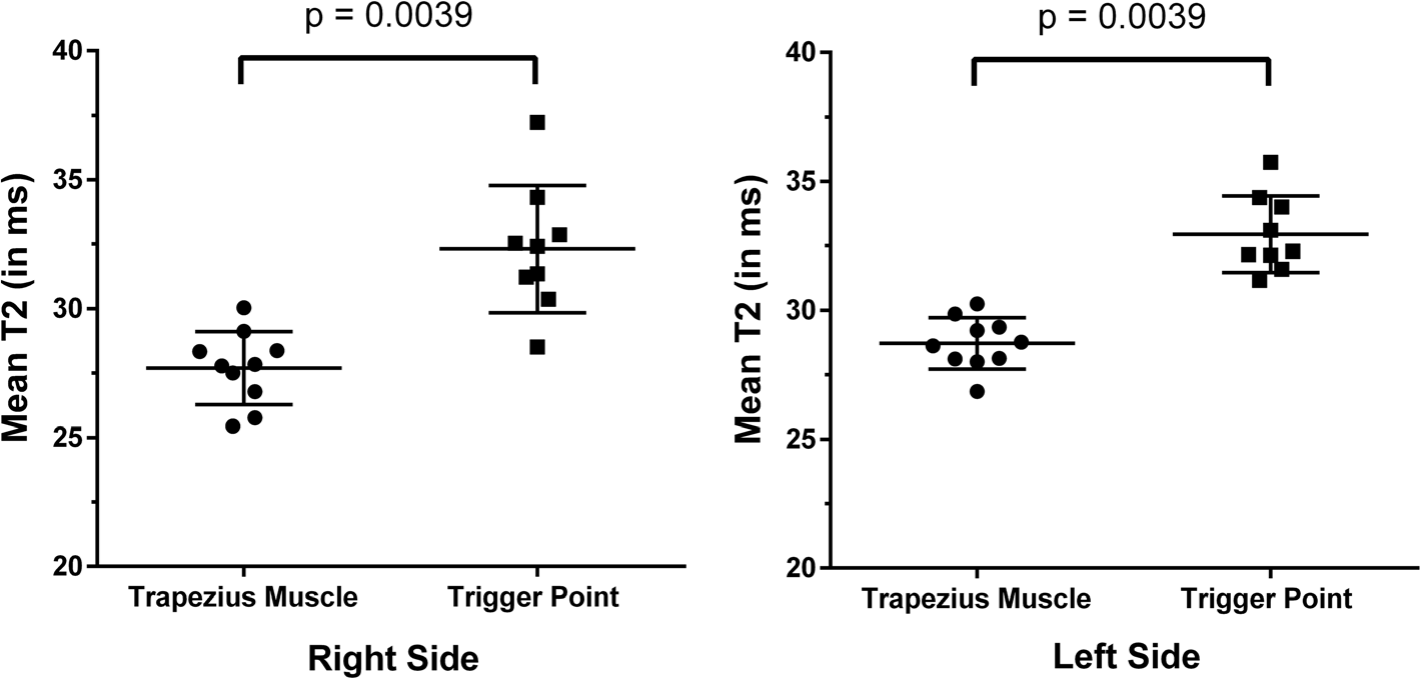
T2 values of the upper trapezius muscles and myofascial trigger points (mTrPs). The graphs show the T2 values (in ms) of each subject derived from the regions of interest (ROIs) enclosing the trapezius muscles and signal alterations attributed to mTrPs of the right and left side, respectively. Measurements in trapezius muscles were obtained in all subjects bilaterally, whereas measurements of T2 values of signal alterations attributed to mTrPs were achieved in nine subjects per side, respectively (due to missing detectable signal alterations in the remaining subjects). Horizontal lines represent the mean with the standard deviation (SD). A statistically significant difference was observed between measurements for both sides, respectively (*p* = 0.0039)

Regarding T2 values of the trapezius muscles, 20 measurements were obtained (ten measurements per side), with an average T2 value of 27.7 ± 1.4 ms (range: 25.5–30.0 ms) for the right-sided and an average T2 value of 28.7 ± 1.0 ms (range: 26.9–30.3 ms) for the left-sided trapezius muscles (Fig. 2). There was no statistically significant difference in these values between sides (*p* = 0.1055).

Concerning T2 values of the signal alterations attributed to mTrPs, nine values were obtained for the right and left side, respectively, with no detected T2 alteration in two different subjects according to evaluation of the color-coded T2 maps. The right-sided signal alterations had an average T2 value of 32.3 ± 2.5 ms (range: 28.5–37.2 ms), the left-sided signal alterations presented a mean T2 value of 33.0 ± 1.5 ms (range: 31.2–35.7 ms; Fig. 2). The difference in T2 values between sides was not statistically significant (*p* = 0.0781).

When comparing the T2 values of the trapezius muscles to the T2 values extracted from the signal alterations attributed to mTrPs of the ipsilateral side, we observed a statistically significant difference for both sides, respectively (*p* = 0.0039 for the right and left side; Fig. 2). Mean T2 values of such signal alterations were higher than the respective T2 values of the trapezius muscles bilaterally in all subjects.

#### Visual image inspection

According to visual image inspection of the T2-weighted DIXON TSE sequence, T2 hyperintensities at the area where signal alterations attributed to mTrPs were identified in the color-coded T2 maps were reported in four subjects regarding the right side and in two subjects regarding the left side (water images; Fig. 1). No correlates of these T2 hyperintensities on water images were found in the corresponding fat images of the T2-weighted DIXON TSE sequence (Fig. 1).

**Fig. 3:**
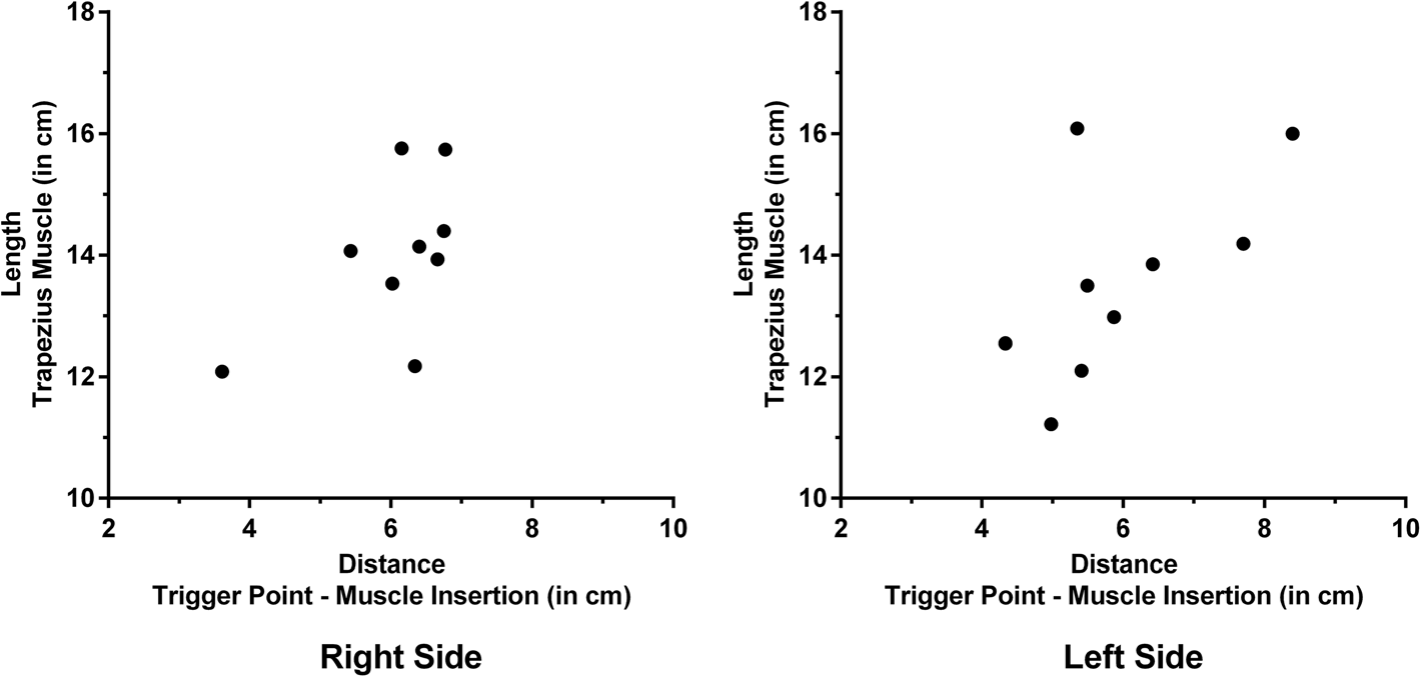
Localization of myofascial trigger points (mTrPs) within the upper trapezius muscles. The graphs intend to provide information on the localization of mTrPs within trapezius muscles by capturing the linearly measured distances between the signal alteration attributed to the mTrP and the muscle insertion of the trapezius muscle (x-axis) in relation to the entire length of the trapezius muscle (y-axis) for both sides, respectively. Measurements of T2 values of signal alterations attributed to mTrPs were achieved in nine subjects per side

According to linear measurements between the muscle insertion and the signal alterations being attributable to mTrPs, we observed a distance of 6.0 ± 0.9 cm (range: 3.6–6.8 cm) for the right and a distance of 6.0 ± 1.2 cm (range: 4.3–8.4 cm) for the left side (Fig. 3).

### Comparison of physical examination and T2 mapping

When taking both sides together, 15 out of the 16 mTrPs according to physical examination were detected in the color-coded T2 maps by means of corresponding signal alterations and elevated T2 values. The remaining mTrP was not detected in color-coded T2 maps. Furthermore, signal alterations on the theoretical connecting line between the capsules were registered three times without previous detection of mTrPs according to physical examination.

## Discussion

This study applied high-resolution MRI with T2 mapping at the level of the upper trapezius muscles for the identification and quantification of active mTrPs in subjects with migraine. When comparing T2 values derived from the trapezius muscles and T2 values of signal alterations attributed to mTrPs, we observed statistically significant differences, with elevated values of such signal alterations when compared to surrounding musculature.

Previous research has shown a high occurrence of mTrPs in subjects with migraine and has linked mTrPs to neck mobility [11–15]. Evidence of an important role of mTrPs of the neck area in the context of migraine is further provided by the finding that migraine attacks can be triggered by manually applied pressure to these points [12, 16]. A widely accepted hypothesis regarding the underlying pathologic mechanism is the concept of the trigemino-cervical complex (TCC) [7, 18, 37]. The TCC is basically characterized by a convergence of nociceptive inputs originating from the neck and shoulder muscles (C1 to C3) and the first branch of the trigeminal nerve in the trigeminal nuclei; thus, it could represent a connecting loop between the central nervous system and peripheral structures beyond, such as the trapezius muscles and its sensory afferences. In line with this hypothesis, migraine has partially been attributed to nociceptive myofascial inputs that increased cortical neuronal excitability, with reported lower pressure pain thresholds of upper trapezius muscles in subjects with migraine when compared to controls without migraine [38, 39]. This suggests that hyperalgesia perceived in neck and shoulder muscles might indeed contribute to the development and/or maintenance of migraine via cervical-to-trigeminal linking and vice versa, with mTrPs potentially playing a key role as morphologically identifiable correlates.

Despite mTrPs seem to be closely associated with migraine, identification and characterization of such points lacks behind. The gold standard for detection of mTrPs is represented by physical examination of muscles and has basically not changed since decades although other techniques, including in-vivo imaging, have developed in the meantime [17, 18]. It seems evident that mere physical examination has to be questioned with respect to reproducibility and reliability, and investigators have indeed shown considerable disagreement during the diagnosis of mTrPs [19, 20]. Efforts using other techniques than physical examination have been undertaken, providing a mixed and heterogeneous picture regarding results. IT has demonstrated disagreement concerning skin temperature patterns in the presence of mTrPs [31]. EMG revealed that endplate noise was more common in mTrPs than in other areas, and intramuscular activity was higher at rest and during contraction at mTrPs when compared with other sites [29, 30]. US should principally be capable of directly visualizing potential alterations in association with mTrPs; however, results on the detectability and characterization of mTrPs are partially contradictory [23–26]. Nevertheless, advanced combinations of US with texture analysis or elastography were reported to successfully differentiate between mTrPs and asymptomatic muscle tissue and to distinguish the type of mTrPs, with mTrPs typically appearing as hypoechoic regions during US examinations [23, 25, 26]. Nevertheless, US has not found the way to a standardized routine procedure and remains rather experimental. Although MRI principally seems the modality of choice regarding imaging of skeletal musculature due to superior soft tissue contrast, the technique has not been in the focus of research on mTrPs, with previous studies showing poor agreement between physicians and imaging raters or being limited regarding generalizability of findings due to small series [21, 22].

High-resolution MRI-based T2 mapping, enabling quantitative and more objective assessments, has not been applied yet. The results of this first study seem promising, with the key finding of significantly elevated T2 values attributable to mTrPs and an accordance between physical examination and imaging regarding detectability in 15 out of the 16 mTrPs defined by manual palpation. Novel developments in the field of MRI make the application of T2 mapping at the level of the upper trapezius muscle possible now: conventional T2 mapping approaches were primarily based on two-dimensional, multi-slice multi-echo spin echo sequences that showed to suffer from the dependence of the T2 quantification on B1 and B0 errors [40, 41]. In contrast, our T2-prepared TSE sequence offers an accurate and fast 3D T2 quantification that has shown to be robust to both B1 and B0 errors even at a challenging region such as the neck and shoulder region where large B0 variations can occur [32]. Superiority of the T2 mapping approach to mere structural imaging is suggested by the finding that only a minority of subjects showed T2 hyperintensities on water images derived from the T2-weighted DIXON TSE sequences according to qualitative, visual image evaluation. No clear corresponding signal alterations were observed in the fat images; thus, we can exclude fatty muscle infiltration or other fat-related structures constituting potential mTrPs. At the current stage, edematous changes might best explain the T2 hyperintensities observed; however, the distinct nature of these changes (e.g., due to chronic inflammation or other causes) yet has to be elucidated.

Our approach might already entail clinical implications. Improved and more objective identification of mTrPs within the upper trapezius muscles may help to guide interventions for the treatment of migraine by targeting these points specifically in the context of the TCC. Remarkably, different invasive and non-invasive intervention approaches were applied to target mTrPs in subjects with migraine [18, 42]; yet, guidance is mostly led by physical examinations. The currently rather limited benefit from such interventions might potentially be enhanced by more tailored applications with knowledge about the presence and exact location of mTrPs, information that can be provided by our approach. Specifically, T2 mapping might define the target region for modulation by repetitive peripheral magnetic stimulation (rPMS), which has shown to relieve pain in subjects with migraine when applied to the upper trapezius muscles [43]. Pre- and post-interventional MRI including T2 mapping could allow correlating improved symptoms to quantitative changes within stimulated muscles and mTrPs in the context of longitudinal study designs. Later follow-up MRI with T2 mapping would further allow monitoring such potential quantitative changes over time, thus allowing to determine whether an intervention has caused longer-lasting or rather transient changes within the examined musculature. Furthermore, other conditions such as fibromyalgia, for instance, have also shown associations with mTrPs [44–46]. While this study focused on subjects with migraine, it appears obvious to evaluate our approach in such conditions as well. Thus, applicability of MRI including T2 mapping might not be restricted to the mTrPs of subjects with migraine, but could also be tested as a more objective, quantitative assessment tool in other diseases known to have close links to mTrPs.

When interpreting the results of our study some limitations have to be considered. First, the small size of the cohort and its constitution of predominantly female subjects limit generalizability of findings at the current stage. However, although only ten subjects were enrolled, we were able to analyze bilateral mTrPs in the majority of subjects, thus increasing the number of total measurements. Second, the lack of a control group consisting of non-migraineurs represents a limitation. Inclusion of such a control group might have allowed to more distinctly link signal alterations attributed to mTrPs to the condition of migraine. Third, we strictly focused on the mTrP per side that showed the highest intensity of referred cranial pain, thus not considering potential other mTrPs. It would be interesting to also extract and evaluate T2 values of these and to incorporate also latent mTrPs in future studies. Fourth, we found one mTrP derived from manual palpation that showed no correlate in color-coded T2 maps and three signal alterations on the theoretical connecting line between the markers that were not previously classified as mTrPs by means of manual palpation. In this context, physical examination to detect mTrPs represents the current gold standard, but has shown shortcomings [19, 20]. Currently, it remains unclear whether T2 mapping is superior to manual palpation and whether physical examination or T2 mapping was correct in these cases. Fifth, it has to be acknowledged that MRI is generally more expensive than one-time physical examination, and T2 mapping is not broadly available yet. Thus, our presented approach might not be directly transferred to clinical routine in all centers dealing with subjects suffering from migraine at the current stage. Future studies including larger cohorts, consisting of both males and females and non-migraineurs as controls, are needed to confirm the results of this study and to further evaluate the potential of T2 mapping in the light of physical examination and in comparison to other point-of-care techniques, particularly US.

## Conclusions

This study is the first to apply quantitative MRI by means of T2 mapping for the identification of mTrPs within upper trapezius muscles in subjects with migraine. Signal alterations in color-coded T2 maps attributed to mTrPs presented with significantly elevated T2 values in comparison to the surrounding musculature bilaterally, and our approach allowed for the detection of mTrPs even in the absence of qualitatively assessed signal alterations. Our approach might challenge the current gold-standard method of physical examination to detect mTrPs, but our initial results have to be confirmed in larger cohorts and by considering also latent mTrPs. Nevertheless, our approach could already allow to verify the local effect of therapeutic approaches to the muscle (e.g., to mTrPs), enable targeted applications to the mTrPs (e.g., physiotherapy, acupuncture, rPMS, or botulinum toxin), and support studies to elucidate further the role of TCC in migraine.

## Abbreviations

3D: Three-dimensional
EMG: Electromyography
FOV: Field of view
IT: Infrared thermography
MPR: Multi-planar reconstruction
MRI: Magnetic resonance imaging
mTrP: Myofascial trigger point
ROI: Region of interest
rPMS: Repetitive peripheral magnetic stimulation
SD: Standard deviation
TCC: Trigemino-cervical complex
TE: Echo time
TR: Repetition time
TSE: Turbo spin echo
US: Ultrasound
